# Supplementary material for: Transcriptional reprogramming from innate immune functions to a pro-thrombotic signature by monocytes in COVID-19
Source: Nat Commun. 2022 Dec 26;13:7947. doi: 10.1038/s41467-022-35638-y (PMC9791976; doi:10.1038/s41467-022-35638-y)
Supplement: Supplementary file 1 — Supplementary information [file 41467_2022_35638_MOESM1_ESM.pdf]

Transcriptional reprogramming from innate immune functions to a pro-thrombotic signature by monocytes in COVID-19

Allison K. Maher[1], Katie L. Burnham[2], Emma M Jones[1], Michelle M. H. Tan[3], Rocel C. Saputil[3], Laury Baillon[1], Claudia Selck[1], Nicolas Giang[1], Rafael Argüello[4], Clio Pillay[3], Emma Thorley[3], Charlotte-Eve Short[1], Rachael Quinlan[1], Wendy S. Barclay[1], Nichola Cooper[3], Graham P. Taylor[1], Emma E. Davenport[2], Margarita Dominguez-Villar[1,*].

[1]Department of Infectious Diseases, Faculty of Medicine, Imperial College London, London, UK, [2]Wellcome Sanger Institute, Wellcome Genome Campus, Hinxton, Cambridge, UK, [3]Department of Immunology and Inflammation, Faculty of Medicine, Imperial College London, UK, [4]Aix Marseille Université, CNRS, INSERM, Centre d'Immunologie de Marseille-Luminy, Marseille, France.

* correspondence to m.dominguez-villar@imperial.ac.uk

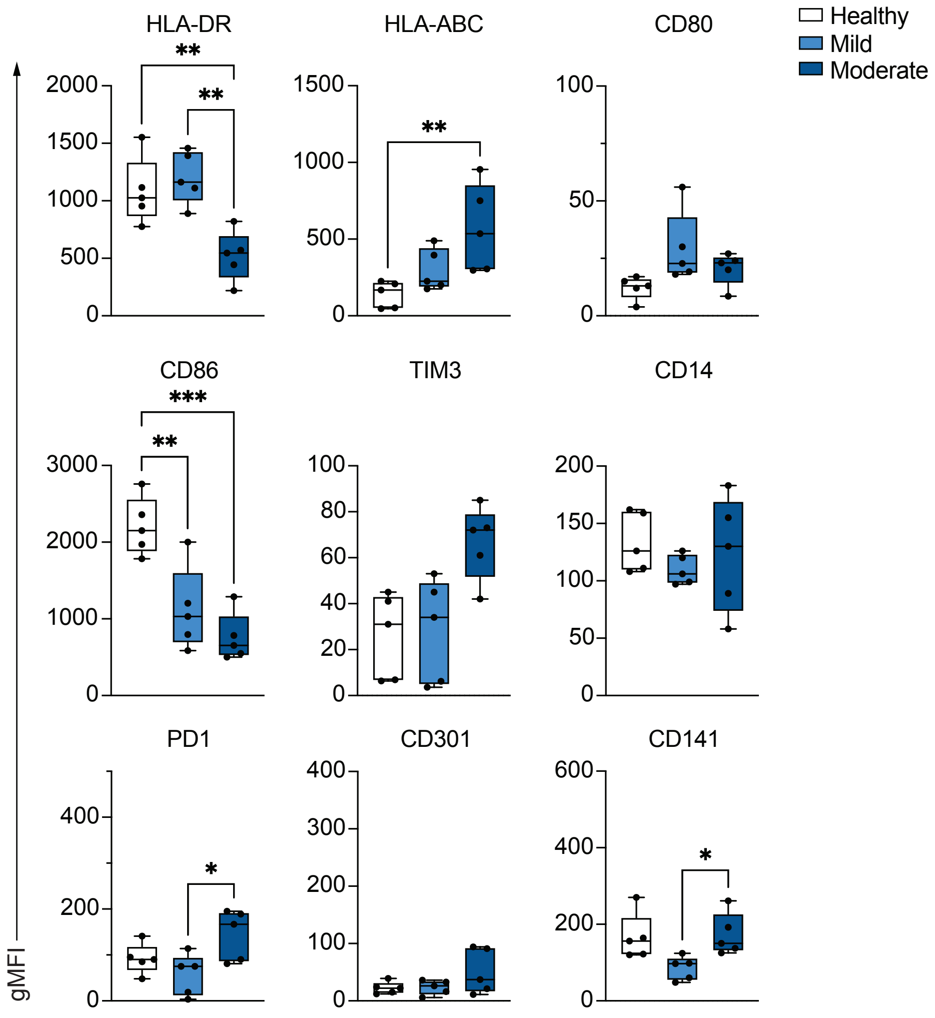

Supplementary Figure 1. Monocyte phenotype in a second cohort of individuals. Box and whiskers plots summarizing the median gMFI of each of the cell surface receptors analyzed (horizontal line inside boxes) in a second cohort of healthy individuals, mild and moderate COVID-19 patients (n=5 participants per group). The box extends from the 25th to the 75th percentile and the whiskers are drawn down to the minimum and up to the maximum gMFI value obtained in each group. One-way ANOVA with Tukey's correction for multiple comparisons. *p<0.05, **p<0.005, ***p<0.001.

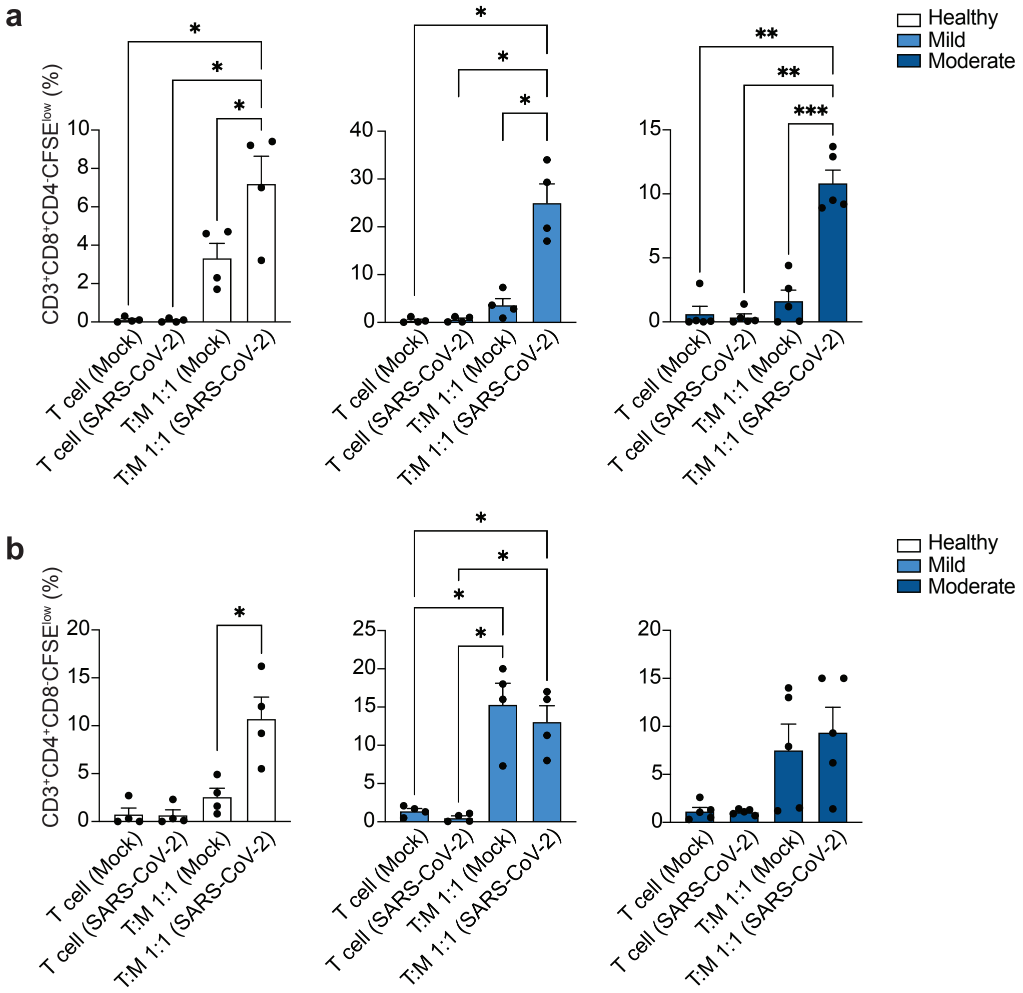

Supplementary Figure 2. CD14⁺ monocytes from COVID-19 patients effectively activate virus-specific CD8⁺ T cells, but not CD4+ T cells. *Ex vivo* isolated CD14⁺ monocytes from healthy individuals (white bars, n=4), mild (light blue, n=4) and moderate COVID-19 patients (dark blue, n=5) were loaded with UV-inactivated SARS-CoV-2 or vehicle (mock) and co-cultured with CFSE-labeled CD3⁺ T cells at a 1:1 monocyte:T cell ratio for 10 days. Isolated T cells incubated with mock or UV-inactivated SARS-CoV-2 served as negative controls for proliferation, Bar plots represent the summary of the percentage of SARS-CoV-2-specific CD8⁺ (**a**) and CD4⁺ (**b**) T cells measured by CFSE dilution and shown as mean+s.e.m. One-way ANOVA with Tukey's correction for multiple comparisons. *p<0.05, **p<0.005, ***p<0.001.

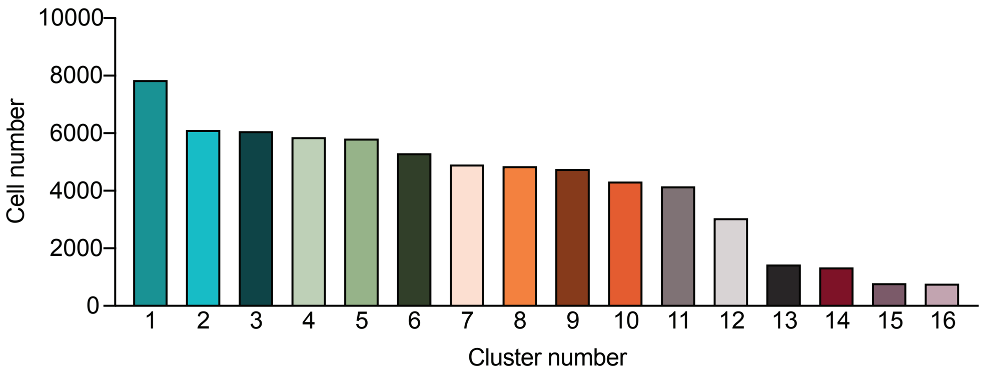

Supplementary Figure 3. Number of cells per cluster identified by Phenograph. Each bar represents the number of cells that each cluster (x axis) contains.

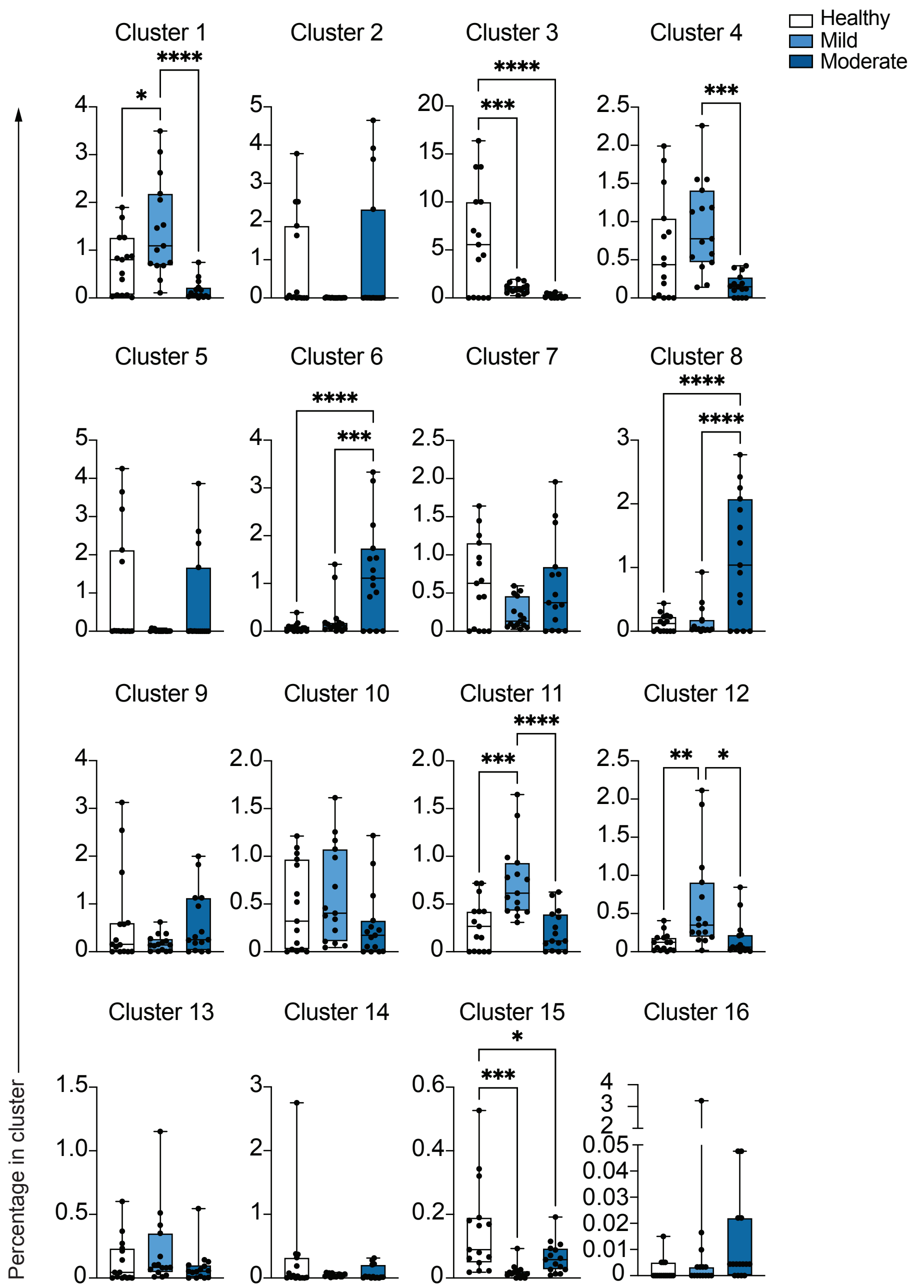

Supplementary Figure 4. Percentage of CD14⁺ monocytes from each clinical group in each Phenograph cluster. Each dot represents an individual, grouped into healthy individuals (white), mild (light blue) and moderate (dark blue) COVID-19 patients (n=15 individuals per group). The boxes extend from the 25th to the 75th percentile and the whiskers are drawn down to the minimum and up to the maximum gMFI values obtained in each group. The horizontal line within each whisker represents the median. One-way ANOVA with Tukey's correction for multiple comparisons. *p<0.05, **p<0.005, ***p<0.001, ****p<0.0005.

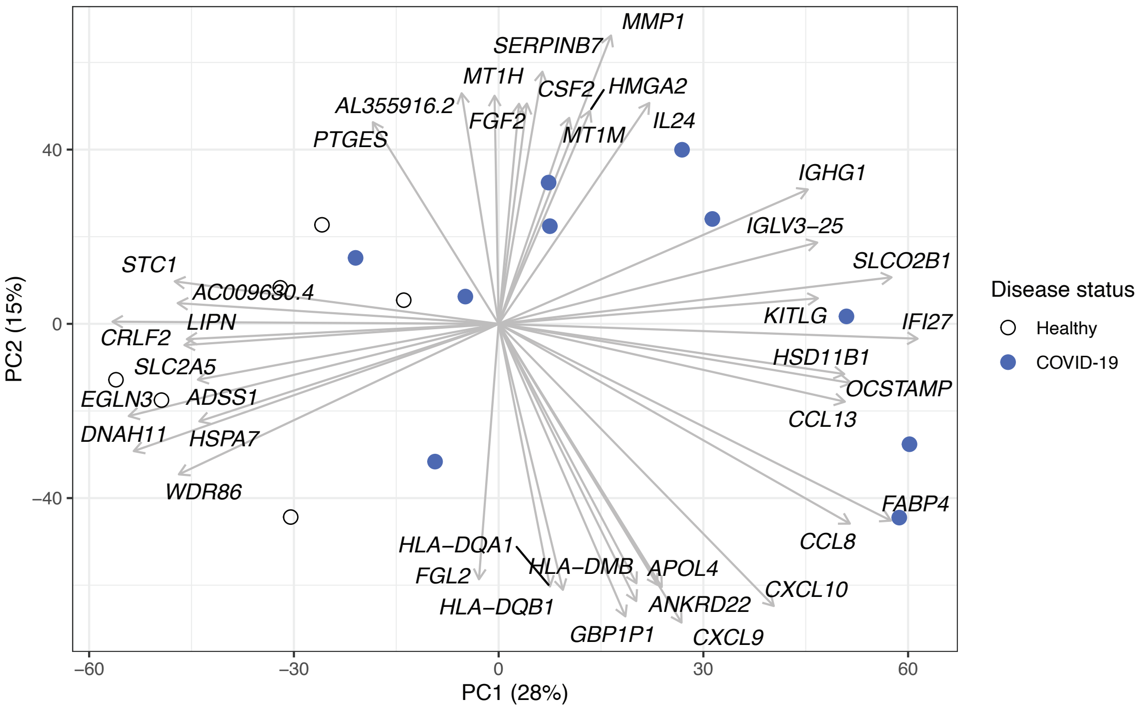

Supplementary Figure 5. Principal Component Analysis (PCA) gene loadings for RNA-seq of *ex vivo* isolated CD14⁺ monocytes from healthy individuals and moderate COVID-19 patients. The features contributing the most to PC1 and PC2 both positively and negatively were identified for healthy individuals (white dots) and COVID-19 patients (blue dots) using gene loadings. The top 10 features for each PC are indicated, with arrows drawn from the origin illustrating their relative weights.

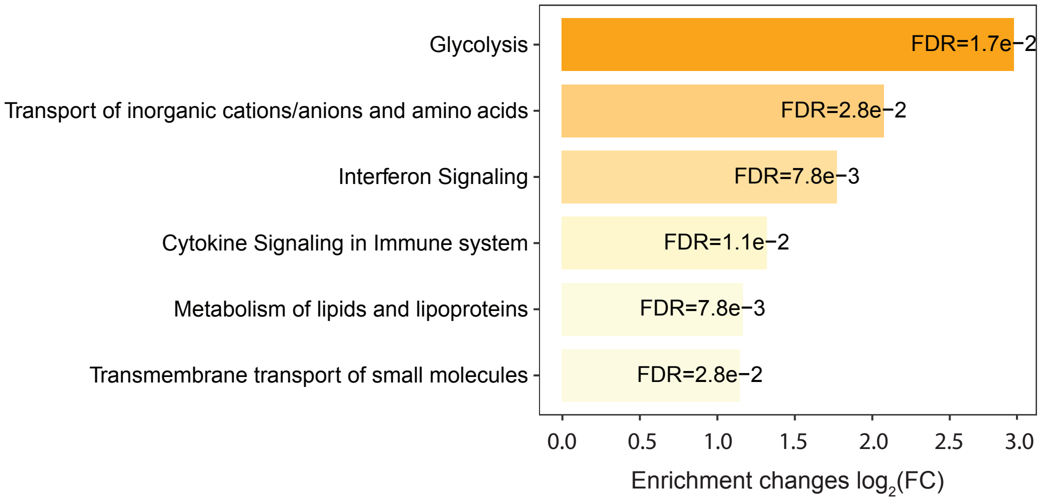

Supplementary Figure 6. Pathway enrichment of COVID-19 monocyte RNA-seq data. Significantly enriched (FDR <0.05) pathways from Reactome for the *ex vivo* COVID-19 *vs.* healthy control monocytes differentially expressed genes are displayed as a bar plot, with the fold enrichment plotted on the x axis (log_2(FC)) and the bars labeled with the adjusted p value, calculated using Fisher's exact test .

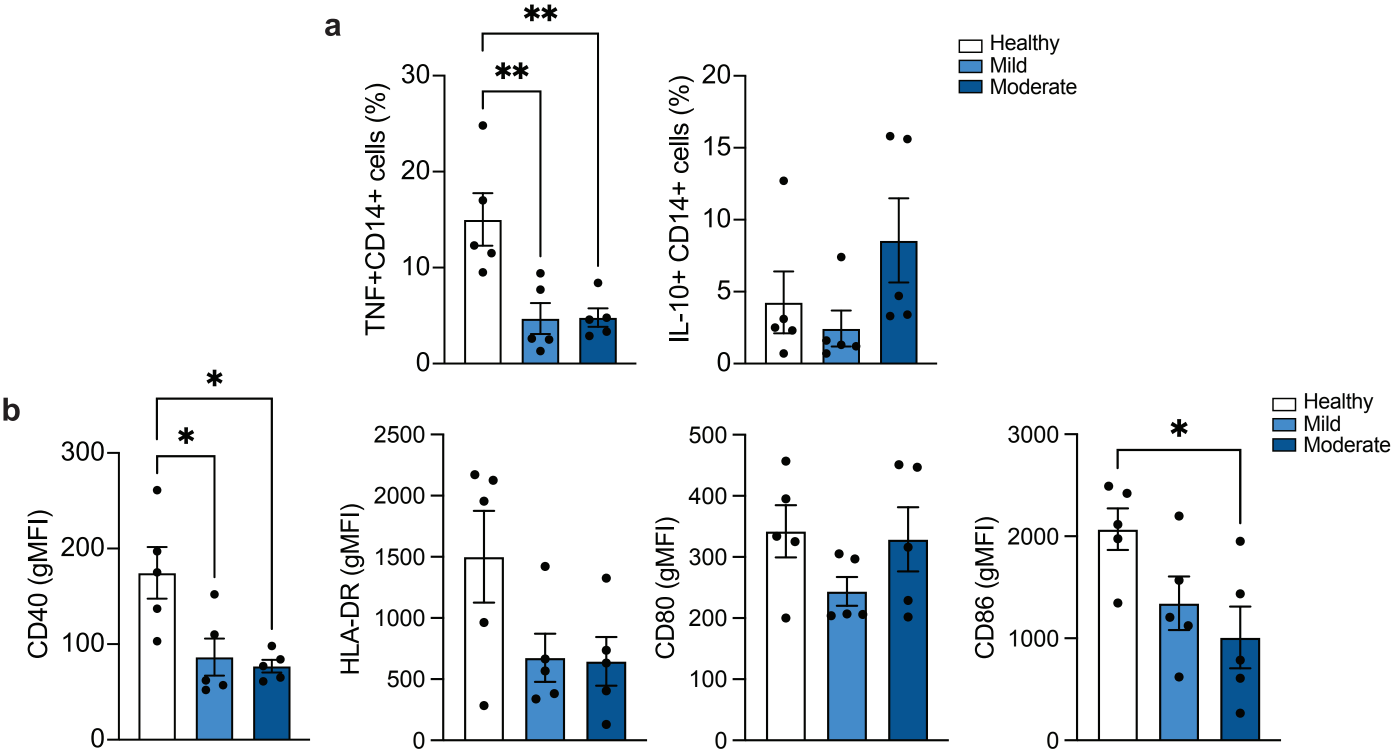

Supplementary Figure 7. Impaired *ex vivo* pathogen sensing by isolated monocytes in COVID-19. $CD14^+$ cells were isolated from total PBMC of healthy individuals (white bars, n=5), mild (light blue bars, n=5) and moderate (dark blue bars, n=5) COVID-19 patients by magnetic sorting and stimulated with UV-inactivated SARS-CoV-2 as in Figure 3. **a**. Bar plots show the summary of percentage of TNF- and IL-10-secreting monocytes in each participant group. **b**. Bar plots represent the summary of HLA-DR, CD40, CD80 and CD86 protein expression (gMFI) in each participant group. Data represented as mean +s.e.m. One-way ANOVA with Tukey's correction for multiple comparisons. *p<0.05, **p<0.005.

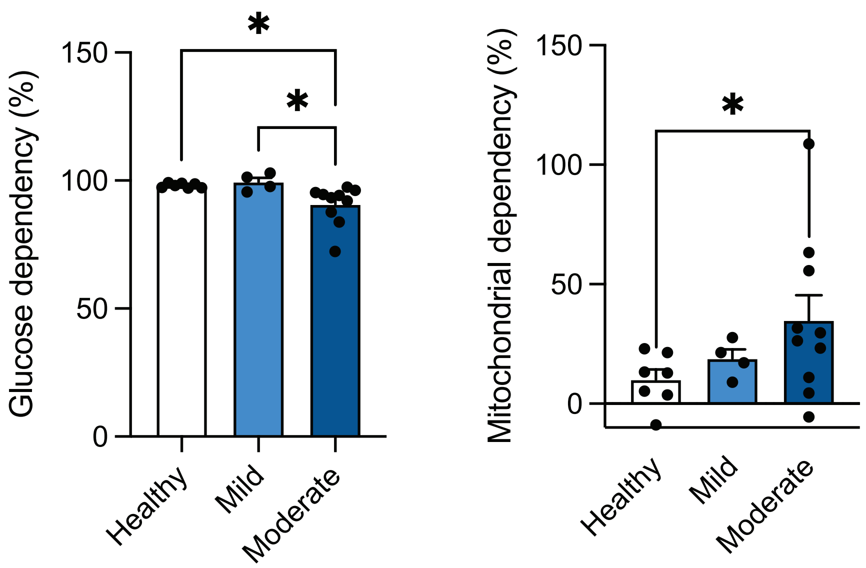

Supplementary Figure 8. Monocyte glucose metabolism and mitochondrial oxidation dependency after LPS stimulation. Glucose dependency and mitochondrial oxidation dependency determined using SCENITH^{TM} in healthy individuals (n=7, white bar), mild (n=4, light blue bar) and moderate (n=9 for glucose dependency and n=10 for mitochondrial dependency, dark blue bar) COVID-19 monocytes after a 18 hour stimulation with 100 ng/ml LPS. Bar plots represent the mean+s.e.m. One-way ANOVA with Tukey's correction for multiple comparisons. *p<0.05.

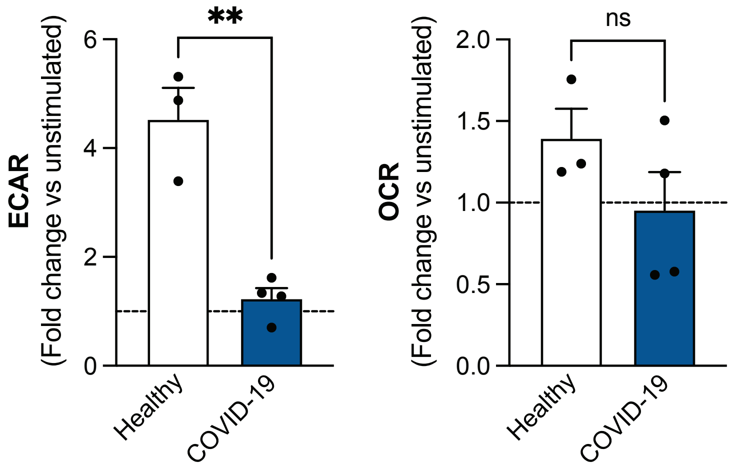

Supplementary Figure 9. Seahorse analysis of activated COVID-19 monocytes. Extracellular acidification rate (ECAR) and oxygen consumption rate (OCR) were measured in sorted $CD14^+$ monocytes from healthy individuals (n=3) and COVID-19 patients (n=4) stimulated or not with 100 ng/ml LPS for 18 hours, shown as mean+s.e.m.. ECAR and OCR shown as fold increase relative to unstimulated controls **$p<0.005$ by unpaired, two-tailed t-test.

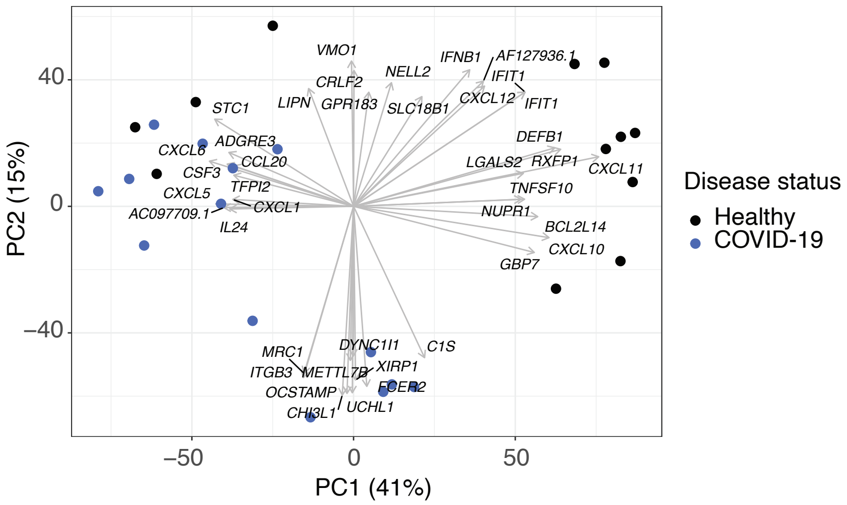

Supplementary Figure 10. Principal Component Analysis (PCA) gene loadings for RNA-seq of SARS-CoV-2-stimulated CD14⁺ monocytes from healthy individuals and moderate COVID-19 patients. The features contributing the most to PC1 and PC2 (both positively and negatively) were identified using gene loadings, and the top 10 features for each PC are indicated, with arrows drawn from the origin illustrating their relative weights.

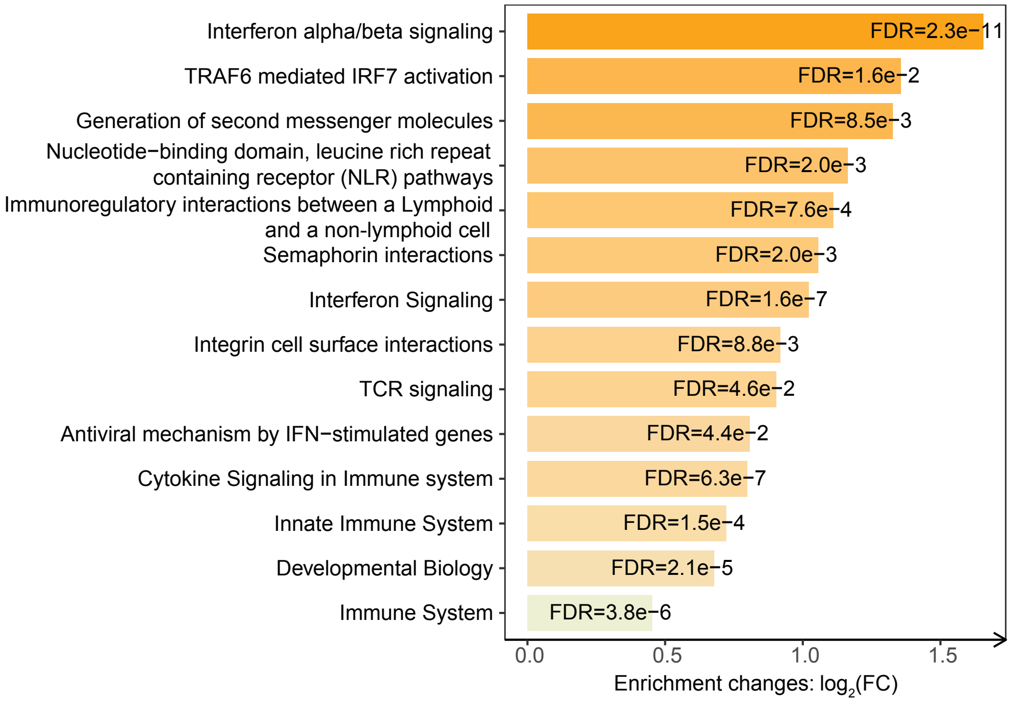

Supplementary Figure 11. Pathway enrichment of SARS-CoV-2-stimulated COVID-19 monocyte RNA-seq data. Significantly enriched (FDR <0.05) pathways from Reactome for SARS-CoV-2-stimulated COVID-19 vs. healthy control monocytes differentially expressed genes are displayed as a bar plot, with the fold enrichment plotted on the x axis (log_2(FC)) and the bars labeled with the adjusted p value, calculated using Fisher's exact test.

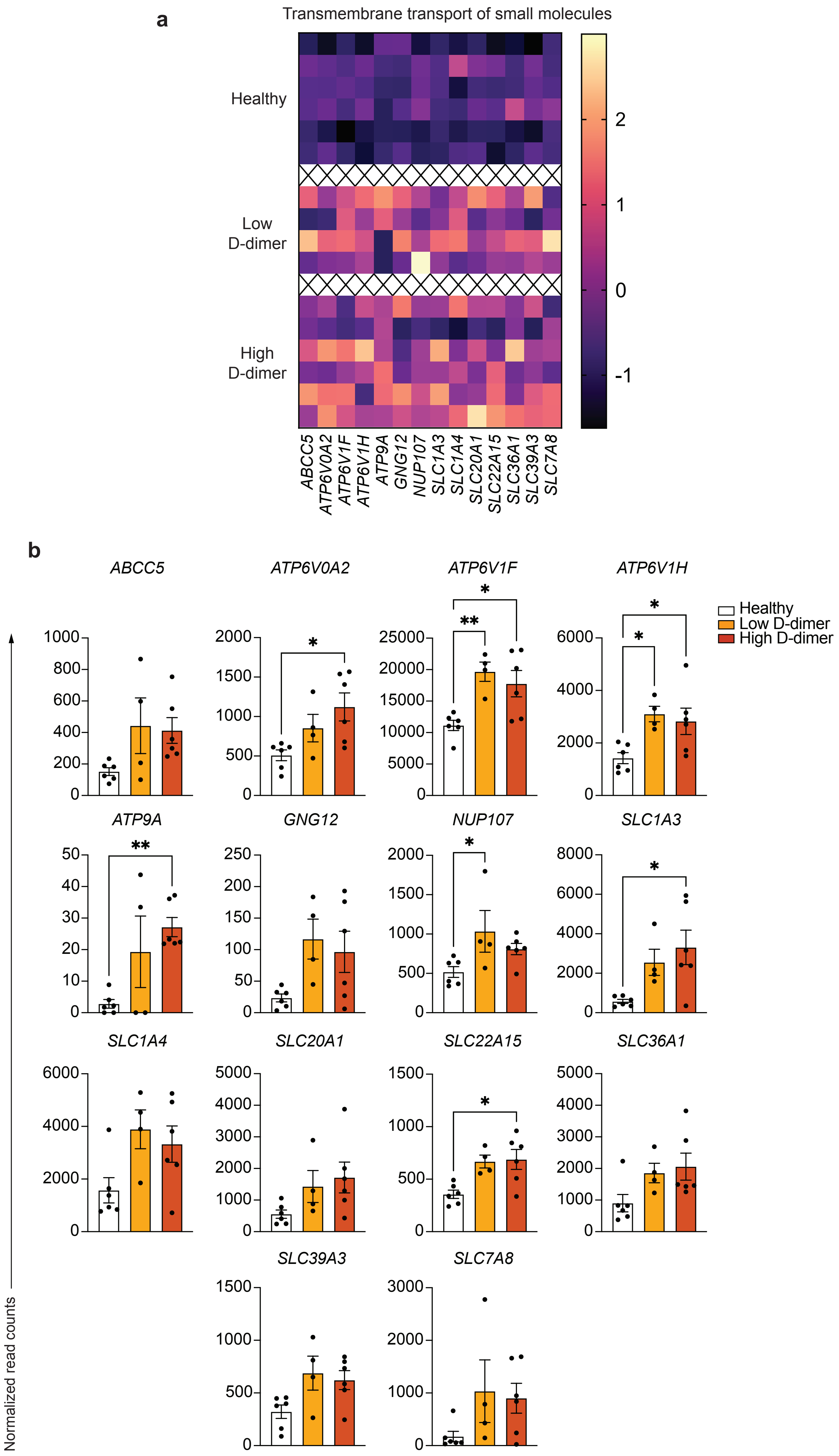
a
Transmembrane transport of small molecules
Healthy
Low D-dimer
High D-dimer
ABCC5
ATP6V0A2
ATP6V1F
ATP6V1H
ATP9A
GNG12
NUP107
SLC1A3
SLC1A4
SLC20A1
SLC22A15
SLC36A1
SLC39A3
SLC7A8
b
Healthy
Low D-dimer
High D-dimer
Normalized read counts
ABCC5
ATP6V0A2
ATP6V1F
ATP6V1H
ATP9A
GNG12
NUP107
SLC1A3
SLC1A4
SLC20A1
SLC22A15
SLC36A1
SLC39A3
SLC7A8

Supplementary Figure 12. **High plasma D-dimer concentration is associated with increased expression of hemostasis-related pathways in *ex vivo* monocyte RNA-seq data**. RNA-seq data from *ex vivo* isolated monocytes from moderate COVID-19 patients were grouped into low and high plasma D-dimer concentrations. **a**. Heatmap of z-score-transformed normalized read counts of significantly upregulated genes in “Transmembrane transport of small molecules” pathway in healthy (n=6), low D-dimer concentration (n=4) and high D-dimer concentration (n=6) moderate COVID-19 monocytes. Gene expression values are scaled by column, and each row represents one individual. **b**. Bar plots show the summary of normalized gene counts of the genes in **a**, shown as mean+s.e.m. One-way ANOVA with Tukey’s correction for multiple comparisons for **b**. *p<0.05, **p<0.005.

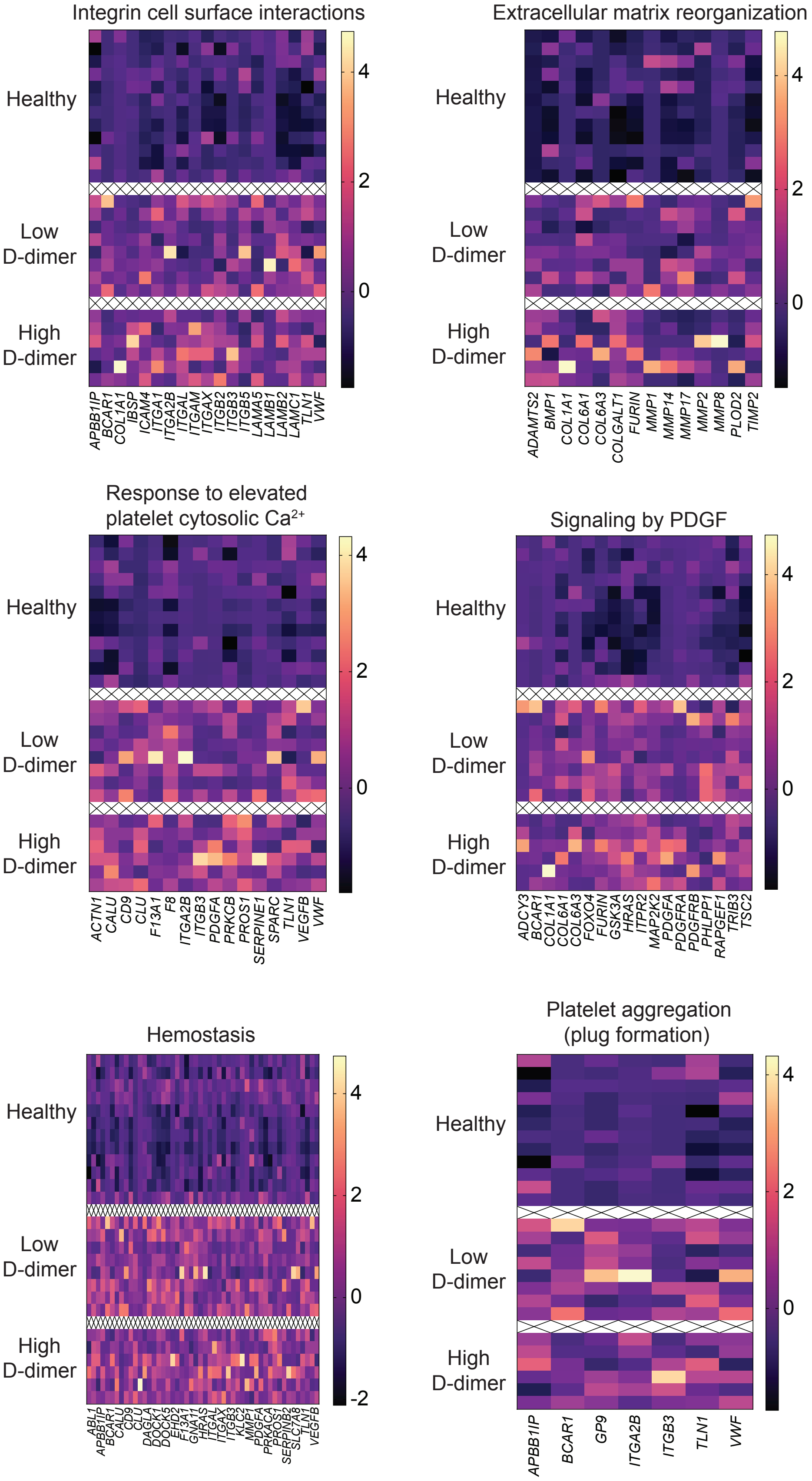
Integrin cell surface interactions
Healthy
Low
D-dimer
High
D-dimer
APBB1IP BCAR1 COL1A1 IBSP ICAM4 ITGA1 ITGA2B ITGAL ITGAM ITGAX ITGB2 ITGB3 ITGB5 LAMA5 LAMB1 LAMB2 LAMC1 TLN1 VWF
4
2
0
Extracellular matrix reorganization
Healthy
Low
D-dimer
High
D-dimer
ADAMTS2 BMP1 COL1A1 COL6A1 COL6A3 COLGALT1 FURIN MMP1 MMP14 MMP17 MMP2 MMP8 PLOD2 TIMP2
4
2
0
Response to elevated
platelet cytosolic Ca2+
Healthy
Low
D-dimer
High
D-dimer
ACTN1 CALU CD9 CLU F13A1 F8 ITGA2B ITGB3 PDGFA PRKCB PROS1 SERPINE1 SPARC TLN1 VEGFB VWF
4
2
0
Signaling by PDGF
Healthy
Low
D-dimer
High
D-dimer
ADCY3 BCAR1 COL1A1 COL6A3 FOXO4 FURIN GSK3A HRAS ITPR2 MAP2K2 PDGFA PDGFRA PDGFRB PHLPP1 RAPGEF1 TRIB3 TSC2
4
2
0
Hemostasis
Healthy
Low
D-dimer
High
D-dimer
ABL1 APBB1IP BCAR1 CALU CD9 CLU DAGLA DOCK1 DOCK5 EHD2 F13A1 GNA11 HRAS ITGAL ITGAX ITGB3 KLC2 MMP1 PDGFA PRKACA PROS1 SERPINB2 SLC7A8 TLN1 VEGFB
4
2
0
-2
Platelet aggregation
(plug formation)
Healthy
Low
D-dimer
High
D-dimer
APBB1IP BCAR1 GP9 ITGA2B ITGB3 TLN1 VWF
4
2
0

Supplementary Figure 13. **Secondary SARS-CoV-2 stimulation increases the expression of pro-thrombotic genes in a D-dimer concentration independent manner**. RNA-seq datasets from SARS-CoV-2-stimulated monocytes from moderate COVID-19 patients were grouped into low and high plasma D-dimer concentrations. Heatmap of z-score-transformed normalized read counts of significantly upregulated genes in “Integrin cell surface interactions”, “Extracellular matrix organization”, “Response to elevated platelet cytosolic Ca^{2+}”, “Signaling by PDGF”, “Hemostasis” and “Platelet aggregation (plug formation)” pathways in healthy (n=12), low D-dimer concentration (n=8) and high D-dimer concentration (n=6) moderate COVID-19 monocytes. Gene expression values are scaled by column, and each row represents one individual.

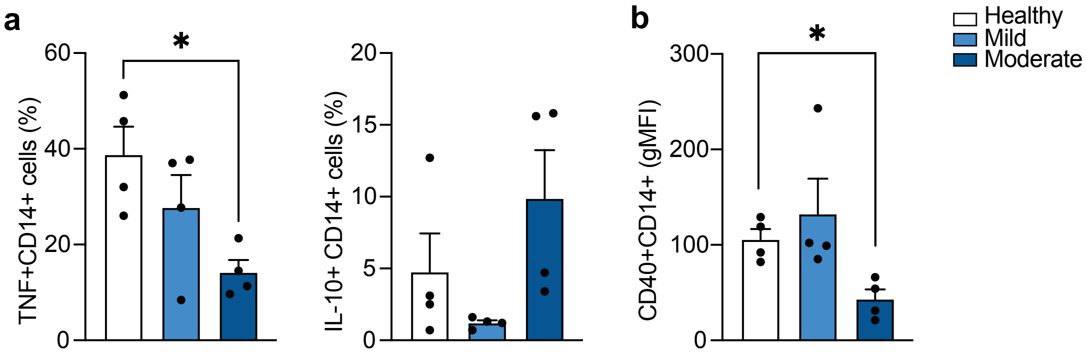

Supplementary Figure 14. Isolated monocytes from moderate COVID-19 patients are hyporesponsive upon secondary LPS stimulation. Monocytes from healthy individuals, mild and moderate COVID-19 patients (n=4 individuals per group) were isolated by CD14 positive magnetic selection and stimulated with 100 ng/ml LPS for 20 hours. **a**. Bar plots show the summary of percentage of TNF- and IL-10-producing $CD14^+$ cells measured by flow cytometry and presented as mean+s.e.m. **b.** Bar plot shows the summary of CD40 expression measured by flow cytometry and plotted as gMFI of $CD14^+$ cells (mean+s.e.m.). One-way ANOVA with Tukey's correction for multiples comparisons in **a** and **b**. *p<0.05, **p<0.005.

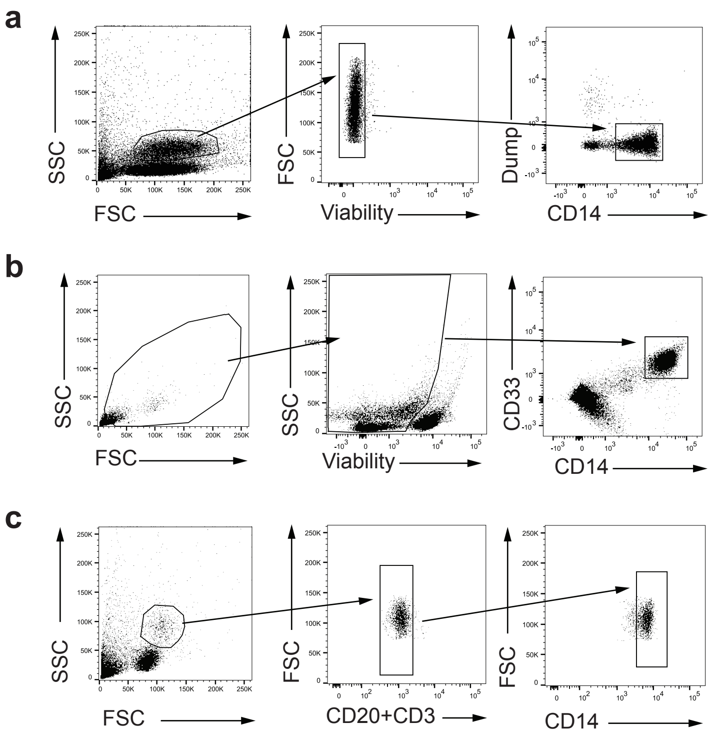

Supplementary Figure 15. Gating strategy for flow cytometry data. **a**. Gating strategy for figures 1a-b, 3c-d, 4a-i and monocyte sort for RNA-seq. Dump channel comprises antibodies to CD3, CD19, CD66b, CD16, and CD56. **b**. Gating strategy for figures 6a-c. **c**. Gating strategy for figures 2e-f, 2h-k, 5g, 5i.

Target	Fluorochrome	Clone	Brand	Catalog number	Dilution
CD14	Pacific Blue	61D3	eBioscience	48-0149-42	1:200
CD14	APC	M5E2	Biolegend	301812	1:200
CD3	PerCP-Cy5.5	UCHT1	BD Biosciences	560835	1:200
CD3	V450	UCHT1	BD Biosciences	560365	1:200
CD1c	PE-Dazzle 594	L161	Biolegend	331531	1:200
CD86	APC	BU63	Biolegend	374208	1:200
CD80	FITC	BB1	BD Pharmigen	555683	1:200
HLA-DR	APC-H7	L243	Biolegend	307617	1:200
CD301	PE	H037G3	Biolegend	354703	1:200
HLA-ABC	BV510	W6/32	Biolegend	311435	1:200
TIM3	SB702	F38-2E2	Invitrogen	67-3109-42	1:200
CD19	PerCP-Cy5.5	HIB19	BD Biosciences	561295	1:200
PD1	PE-Cy7	EH12.2H7	Biolegend	329917	1:200
CD16	AF700	3G8	BD	557920	1:200
CD16	PerCP-Cy5.5	B73.1	Biolegend	360712	1:200
CD141	BV785	M80	Biolegend	344115	1:200
CD40	Alexa Fluor 700	5C3	Biolegend	334328	1:200
TNF	BV510	Mab11	Biolegend	502950	1:200
IL-10	BV412	JES3-907	ThermoFisher Scientific	48-7108-42	1:200
Puromycin	APC	R4743L-E8	Gift from Dr Rafael Argüello	N/A	1:600
CD41	PE	HIP8	Biolegend	303706	1:100
CD33	APC		BD Biosciences	340474	1:200
CD20	PerCP-Cy5.5	H1	BD Biosciences	558021	1:200
Phosphor-IRF3 (Ser 396)	AF488	Polyclonal	Bioss	bs-3195R-A488	1:666
Phospho-NFkB p65 (Ser 529)	PE-Cy7	K10-895.12.50	BD Biosciences	6273506	1:50
IkBa	PE	3D6C02	Biolegend	662412	1:200
CD4	PE-Dazzle 594	RPA-T4	Biolegend	300548	1:200
CD4	BV605	RPA-T4	Biolegend	300555	1:200
CD4	FITC	RPA-T4	Biolegend	300506	1:200
CD8	PE-Dazzle 594	RPA-T8	Biolegend	301058	1:200
CD56	PerCP-Cy5.5	5.1H11	Biolegend	362526	1:200
CD66b	PerCP-Cy5.5	QA17A51	Biolegend	396914	1:200

Table 1. Antibodies used in the study.
