# Supplementary material for: Transcriptional reprogramming from innate immune functions to a pro-thrombotic signature by monocytes in COVID-19
Source: Nat Commun. 2022 Dec 26;13:7947. doi: 10.1038/s41467-022-35638-y (PMC9791976; doi:10.1038/s41467-022-35638-y)
Supplement: Supplementary file 3 — Reporting Summary [file 41467_2022_35638_MOESM3_ESM.pdf]

nature portfolio

Corresponding author(s): Margarita Dominguez-Villar

Last updated by author(s): 25/11/2022

Reporting Summary

Nature Portfolio wishes to improve the reproducibility of the work that we publish. This form provides structure for consistency and transparency in reporting. For further information on Nature Portfolio policies, see our Editorial Policies and the Editorial Policy Checklist.

Statistics

For all statistical analyses, confirm that the following items are present in the figure legend, table legend, main text, or Methods section.

n/a	Confirmed	
☐	☒	The exact sample size (n) for each experimental group/condition, given as a discrete number and unit of measurement
☐	☒	A statement on whether measurements were taken from distinct samples or whether the same sample was measured repeatedly
☐	☒	The statistical test(s) used AND whether they are one- or two-sided<br>*Only common tests should be described solely by name; describe more complex techniques in the Methods section.*
☐	☒	A description of all covariates tested
☐	☒	A description of any assumptions or corrections, such as tests of normality and adjustment for multiple comparisons
☐	☒	A full description of the statistical parameters including central tendency (e.g. means) or other basic estimates (e.g. regression coefficient) AND variation (e.g. standard deviation) or associated estimates of uncertainty (e.g. confidence intervals)
☐	☒	For null hypothesis testing, the test statistic (e.g. F, t, r) with confidence intervals, effect sizes, degrees of freedom and P value noted<br>*Give P values as exact values whenever suitable.*
☒	☐	For Bayesian analysis, information on the choice of priors and Markov chain Monte Carlo settings
☒	☐	For hierarchical and complex designs, identification of the appropriate level for tests and full reporting of outcomes
☒	☐	Estimates of effect sizes (e.g. Cohen's d, Pearson's r), indicating how they were calculated

Our web collection on statistics for biologists contains articles on many of the points above.

Software and code

Policy information about availability of computer code

Data collection	There was no code involved in data collection. Flow cytometry data acquisition and cell sorting were performed using Diva software (BD Bioscciences, version 8.0).
Data analysis	STAR (v2.7.3), featureCounts (v2.0), FastQC (v1.0.0), RNA-SeQC (v1.0), R (v4.1), DESeq2 (v1.38.1), Pheatmap (v1.0.12), limma (v2.9.8). Open source codes used in this work for RNA-seq analysis have been listed in the materials and methods with their corresponding citations to the original publications

For manuscripts utilizing custom algorithms or software that are central to the research but not yet described in published literature, software must be made available to editors and reviewers. We strongly encourage code deposition in a community repository (e.g. GitHub). See the Nature Portfolio guidelines for submitting code & software for further information.

Data

Policy information about availability of data

All manuscripts must include a data availability statement. This statement should provide the following information, where applicable:

- Accession codes, unique identifiers, or web links for publicly available datasets
- A description of any restrictions on data availability
- For clinical datasets or third party data, please ensure that the statement adheres to our policy

Reference genome GRCh38.99, gene expression data on sepsis patients (GSE46955), endotoxin tolerance signature (microarray data on in vitro LPS-stimulated monocytes, datasets GSE15219 and GSE2224879). The RNA-seq datasets have been uploaded to the EGA portal and the codes are pending.

Field-specific reporting

Please select the one below that is the best fit for your research. If you are not sure, read the appropriate sections before making your selection.

- [x] Life sciences
- [ ] Behavioural & social sciences
- [ ] Ecological, evolutionary & environmental sciences

For a reference copy of the document with all sections, see nature.com/documents/nr-reporting-summary-flat.pdf

Life sciences study design

All studies must disclose on these points even when the disclosure is negative.

Sample size	No a priori sample size calculation was performed, and experiments were carried out using the totality of available samples from COVID19 patients and healthy individuals.
Data exclusions	No data were excluded from the analyses
Replication	Replication of monocyte stimulation with SARS-CoV-2 and cytokine and receptor expression by FACS was performed with those samples for which we had sufficient cell numbers (n=7 samples in 3 independent experiments for replication). All replication experiments were successful
Randomization	Participants were allocated in comparison groups based on clinical information (positivity for COVID19 testing and severity determined by clinicians based on the WHO severity score).
Blinding	Investigators were not blinded to study allocation. Samples from healthy participants were identified by codes that were different from those of COVID19 patients.

Reporting for specific materials, systems and methods

We require information from authors about some types of materials, experimental systems and methods used in many studies. Here, indicate whether each material, system or method listed is relevant to your study. If you are not sure if a list item applies to your research, read the appropriate section before selecting a response.

Materials & experimental systems

n/a	Involved in the study	
	x	Antibodies
	x	Eukaryotic cell lines
x		Palaeontology and archaeology
x		Animals and other organisms
	x	Human research participants
x		Clinical data
x		Dual use research of concern

Methods

n/a	Involved in the study	
x		ChIP-seq
	x	Flow cytometry
x		MRI-based neuroimaging

Antibodies

Antibodies used

Target, Fluorochrome, Clone, Brand, Catalog number, Dilution
CD14 Pacific Blue 61D3 eBioscience 48-0149-42 1:200
CD14 APC M5E2 Biolegend 301812 1:200
CD3 PerCP-Cy5.5 UCHT1 BD Biosciences 560835 1:200
CD3 V450 UCHT1 BD Biosciences 560365 1:200
CD1c PE-Dazzle 594 L161 Biolegend 331531 1:200
CD86 APC BU63 Biolegend 374208 1:200
CD80 FITC BB1 BD Pharmigen 555683 1:200
HLA-DR APC-H7 L243 Biolegend 307617 1:200
CD301 PE H037G3 Biolegend 354703 1:200
HLA-ABC BV510 W6/32 Biolegend 311435 1:200
TIM3 SB702 F38-2E2 Invitrogen 67-3109-42 1:200
CD19 PerCP-Cy5.5 HIB19 BD Biosciences 561295 1:200
PD1 PE-Cy7 EH12.2H7 Biolegend 329917 1:200
CD16 AF700 3G8 BD 557920 1:200
CD16 PerCP-Cy5.5 B73.1 Biolegend 360712 1:200
CD141 BV785 M80 Biolegend 344115 1:200
CD40 Alexa Fluor 700 5C3 Biolegend 334328 1:200
TNF BV510 Mab11 Biolegend 502950 1:200

IL-10 BV412 JES3-907 ThermoFisher Scientific 48-7108-42 1:200
Puromycin APC R4743L-E8 Gift from Dr Rafael Argüello N/A 1:600
CD41 PE HIP8 Biolegend 303706
1:100
CD33 APC BD Biosciences 340474 1:200
CD20 PerCP-Cy5.5 H1 BD Biosciences 558021 1:200
Phosphor-IRF3 (Ser 396) AF488 Polyclonal Bioss
bs-3195R-A488 1:666
Phospho-NFkB p65 (Ser 529) PE-Cy7 K10-895.12.50 BD Biosciences 6273506 1:50
IkBa PE 3D6C02 Biolegend 662412 1:200
CD4 PE-Dazzle 594 RPA-T4 Biolegend 300548 1:200
CD4 BV605 RPA-T4 Biolegend 300555 1:200
CD4 FITC RPA-T4 Biolegend 300506 1:200
CD8 PE-Dazzle 594 RPA-T8 Biolegend 301058 1:200
CD56 PerCP-Cy5.5 5.1H11 Biolegend 362526 1:200
CD66b PerCP-Cy5.5 QA17A51 Biolegend 396914 1:200

Validation

All antibodies are commercially available and validation experiments can be found on their corresponding manufacturer websites. Moreover, all antibodies have been previously used to identify the targets examined in this work in published papers.

Eukaryotic cell lines

Policy information about cell lines

Cell line source(s)

Vero-E6 (Wendy S Barclay;s laboratory), MRC-5 (gift from Dr Robert White, Imperial College London), BSC-1 (Public Health England), LLCMK2 (Public Health England)

Authentication

None of the cell lines underwent any specific procedures for their authentication in our laboratory

Mycoplasma contamination

All the cell lines tested negative for mycoplasma contamination

Commonly misidentified lines
(See ICLAC register)

N/A

Human research participants

Policy information about studies involving human research participants

Population characteristics

The population characteristics of the participants is included in Supplementary Table 1.

Recruitment

Healthy donors (WHO 0) were Imperial College staff with no prior diagnosis of or recent symptoms consistent with COVID-19, and where possible, were matched in age and sex distribution with COVID-19 patients.

Blood samples from the COVID-19 patients examined in this work come from two different studies. COVIDITY study is a prospective observational serial sampling study of whole blood to observe the evolution of SARS-CoV-2 infection to characterize the host response to infection over time in peripheral blood (ethics approval obtained from the Health Research Authority, South Central Oxford C Research Ethics Committee). The population of study were >18 year old patients and/or staff at Imperial College Healthcare NHS Trust/Imperial College London with confirmed COVID-19 from a positive SARS-CoV-2 RT-PCR testing from NHS laboratories or Public Health England. Samples were taken 3-14 days after symptom initiation and were classified as 1 or 2 disease severity.
Samples from patients with moderate COVID-19 admitted to hospitals in London (Hammersmith Hospital, Charing Cross Hospital, Saint Mary's Hospital) and eligible to participate in the MATIS trial (NCT04581954) provided consent (ethics approval by the Health Research Authority, London-Surrey Borders Research Ethics Committee) and blood was collected 3-14 days after disease onset and 0-2 days after hospitalization and positive PCR, and before study treatment initiation. Moderate patients displayed mild of moderate COVID-19 pneumonia, defined as grade 3 or 4 WHO severity. Samples were collected from March 2020 to February 2021 and none of the participants had received a COVID-19 vaccine.

No potential self-selection bias or other biases have been identified.

Ethics oversight

Blood samples from the COVID-19 patients examined in this work come from two different studies. COVIDITY study is a prospective observational serial sampling study of whole blood to observe the evolution of SARS-CoV-2 infection to characterize the host response to infection over time in peripheral blood (ethics approval obtained from the Health Research Authority, South Central Oxford C Research Ethics Committee). The population of study were >18-year-old patients and/or staff at Imperial College Healthcare NHS Trust/Imperial College London with confirmed COVID-19 from a positive SARS-CoV-2 RT-PCR test from NHS laboratories or Public Health England.

Note that full information on the approval of the study protocol must also be provided in the manuscript.

Flow Cytometry

Plots

Confirm that:

- [x] The axis labels state the marker and fluorochrome used (e.g. CD4-FITC).
- [x] The axis scales are clearly visible. Include numbers along axes only for bottom left plot of group (a 'group' is an analysis of identical markers).
- [x] All plots are contour plots with outliers or pseudocolor plots.
- [x] A numerical value for number of cells or percentage (with statistics) is provided.

Methodology

Sample preparation	Peripheral blood mononuclear cells were isolated from blood of participants and used in the experiments. For those instances where isolated monocytes were used, classical CD14+ monocytes were sorted as CD3-CD19-CD14+CD16-. Propidium iodide was used to discard dead cells.
Instrument	Monocytes were sorted on a FACS Aria III (BD). FACS analysis was performed on a BD LSR Fortessa.
Software	Data was collected using Diva software and analyzed in FlowJo (Version 10.8)
Cell population abundance	The purity of samples post sort were tested after sorting using the same markers described above and in all cases they were >97%
Gating strategy	Gating strategy for monocyte populations were achieved by gating on the antigen-presenting cell population on FSC vs SSC, live cells (negative for viability dyes, Thermo Fisher), and CD3-CD19-CD14+CD16-CD56-CD66b-.

- [x] Tick this box to confirm that a figure exemplifying the gating strategy is provided in the Supplementary Information.
