# Supplementary material for: Transcriptional reprogramming from innate immune functions to a pro-thrombotic signature by monocytes in COVID-19
Source: Nat Commun. 2022 Dec 26;13:7947. doi: 10.1038/s41467-022-35638-y (PMC9791976; doi:10.1038/s41467-022-35638-y)
Supplement: Supplementary file 4 — Description of Additional Supplementary Files [file 41467_2022_35638_MOESM4_ESM.docx]

**Description of Additional Supplementary Files**

File Name: Supplementary Data 1

Description: Participant characteristics

File Name: Supplementary Data 2

Description: Percentage of cells per cluster in each study group

File Name: Supplementary Data 3

Description: Pathway enrichment results of all DE genes from unstimulated COVID19 vs unstimulated healthy monocytes

File Name: Supplementary Data 4

Description: Pathway enrichment results using upregulated DE genes from unstimulated COVID19 vs unstimulated healthy monocytes

File Name: Supplementary Data 5

Description: Pathway enrichment results of all downregulated DE genes from unstimulated COVID19 vs unstimulated healthy monocytes

File Name: Supplementary Data 6

Description: Enriched pathways using DEG on stimulated COVID-19 monocytes vs. stimulated healthy monocytes

File Name: Supplementary Data 7

Description: Pathway enrichment results of upregulated DE genes from SARS-CoV-2-stimulated COVID19 vs SARS-CoV-2-stimulated healthy monocytes

File Name: Supplementary Data 8

Description: Pathway enrichment results of downregulated DE genes from SARS-CoV-2-stimulated COVID19 vs SARS-CoV-2-stimulated healthy monocytes

File Name: Supplementary Data 9

Description: Plasma D-dimer concentration at the time of blood draw of the participants for whom RNA-seq samples were prepared.
